# Iron Deficiency Anemia Among Pediatric Celiac Disease Patients at the Armed Forces Hospital Southern Region: Prevalence, Predictors, and Outcomes

**DOI:** 10.7759/cureus.103482

**Published:** 2026-02-12

**Authors:** Khalid Asiri, Badriah G Alasmari, Ehab Hanafy, Sarah Al-Barraq, Shady Wafa, Shami Abdullah Aloqayli, Faris Alsaedi

**Affiliations:** 1 Pediatric Gastroenterology, Armed Forces Hospital Southern Region, Khamis Mushait, SAU; 2 Pediatrics, Armed Forces Hospital Southern Region, Khamis Mushait, SAU; 3 Oncology Center, Armed Forces Hospital Southern Region, Khamis Mushait, SAU; 4 Clinical Nutrition, Armed Forces Hospital Southern Region, Khamis Mushait, SAU

**Keywords:** celiac disease, extraintestinal manifestations, gluten-free diet, growth disorders, iron deficiency anemia, malabsorption, pediatrics, saudi arabia

## Abstract

Background

Celiac disease (CD) is a common immune-mediated enteropathy in children and is frequently associated with iron deficiency anemia (IDA), which may be an initial or prominent extraintestinal manifestation and, in some cases, the presenting feature of the disease. While IDA is well recognized in pediatric CD, data regarding its prevalence, clinical correlates, and longitudinal outcomes in children from southern Saudi Arabia remain limited, particularly in tertiary care settings.

Objectives

The primary objective was to determine the prevalence of IDA at the time of CD diagnosis in children. The secondary objectives were to assess the persistence of IDA at one and two years following initiation of a gluten-free diet and to evaluate demographic, clinical, and treatment-related factors associated with its presence and persistence.

Methods

A retrospective cohort study was conducted at the Armed Forces Hospital - Southern Region, including children and adolescents aged 1-18 years with confirmed CD diagnosed between January 2016 and December 2023. Demographic characteristics, clinical presentation, diagnostic modality, iron therapy details, and hematologic outcomes were extracted from medical records. Associations between patient characteristics and IDA were analyzed using univariable and multivariable logistic regression models.

Results

Ninety-seven children with CD were included, of whom 59 (60.8%) were females. IDA was present at diagnosis in 25 (25.8%) patients. No statistically significant associations were identified between IDA and age at diagnosis, gender, short stature, poor weight gain, or syndromic status. Most patients did not require iron supplementation; among those treated, oral iron was administered once daily for three months, typically using standard pediatric weight-based dosing (approximately 3-6 mg/kg/day of elemental iron). Resolution of IDA occurred in 68.0% of affected children at one year following gluten-free diet initiation and increased to 88.0% by two years. Multivariable analyses demonstrated no significant predictors for either the presence of IDA at diagnosis or its persistence at one-year follow-up.

Conclusions

IDA affected approximately one quarter of children at the time of CD diagnosis in this cohort; however, given the high background prevalence of nutritional iron deficiency in pediatric populations, anemia cannot be attributed exclusively to CD in all cases. The majority of affected children demonstrated hematologic recovery following adherence to a gluten-free diet, with progressive improvement over time, supporting a disease-related contribution in a substantial proportion of patients. No demographic, clinical, or treatment-related factors were independently associated with either the presence or persistence of anemia. These findings underscore the importance of routine anemia screening at diagnosis and continued hematologic monitoring during follow-up in children with CD.

## Introduction

Celiac disease (CD) is a chronic, immune-mediated enteropathy precipitated by dietary gluten exposure in genetically predisposed individuals and characterized by inflammation and villous atrophy of the small intestinal mucosa. It represents one of the most common chronic gastrointestinal disorders in childhood, with a global prevalence estimated between 0.5% and 1% in pediatric populations [1]. Advances in serologic screening and increased awareness have led to a substantial rise in diagnosed cases; however, the clinical spectrum of CD continues to evolve, with many children presenting with non-classical or extraintestinal manifestations.

Among these manifestations, iron deficiency anemia (IDA) is particularly prominent and clinically relevant. IDA may occur as a direct consequence of impaired iron absorption resulting from duodenal mucosal damage, chronic inflammation, and altered iron metabolism [2,3]. Importantly, anemia may be the sole presenting feature of CD, especially in patients lacking overt gastrointestinal symptoms, leading to diagnostic delays if CD is not actively considered during anemia evaluation [4].

Pediatric studies have reported a wide prevalence range of IDA in children with CD, varying from 10% to nearly 50%, depending on age, disease severity, diagnostic criteria, and population characteristics [5,6]. The pathophysiology of IDA in CD is multifactorial. Beyond villous atrophy and reduced absorptive surface area, ultrastructural abnormalities of enterocytes, persistent low-grade inflammation, and dysregulation of iron transport proteins contribute to impaired iron homeostasis [7,8]. These mechanisms may persist even after apparent mucosal healing, explaining cases of prolonged or refractory anemia despite adherence to a gluten-free diet (GFD).

The institution of a strict GFD remains the cornerstone of CD management and is generally associated with the gradual normalization of hemoglobin and iron indices [9]. Nevertheless, several studies have documented incomplete or delayed resolution of IDA, with 10-20% of patients continuing to exhibit anemia one to two years after dietary treatment [6,10].

Factors proposed to influence anemia persistence include age at diagnosis, severity of mucosal damage, delayed diagnosis, coexisting autoimmune or syndromic conditions, and variability in iron supplementation practices [11]. However, findings across studies remain inconsistent, and predictors of anemia persistence are not clearly defined, particularly in pediatric cohorts.

In Saudi Arabia, CD is increasingly recognized, yet regional data, especially from the southern region, remain scarce. Most available studies originate from central or western regions and often focus on prevalence rather than longitudinal outcomes or hematologic recovery [12]. The absence of robust local data from tertiary centers limits the development of evidence-based screening strategies and follow-up protocols for children with CD and IDA.

Understanding the burden, clinical correlates, and outcomes of IDA in pediatric CD within a local healthcare context is essential for optimizing early diagnosis, guiding iron therapy, and preventing long-term complications such as impaired growth, neurocognitive effects, and reduced quality of life. Accordingly, the primary objective of this study was to determine the prevalence of IDA at the time of CD diagnosis among children treated at a tertiary care center in southern Saudi Arabia. The secondary objectives were to evaluate the persistence of IDA following initiation of a GFD and to examine demographic and clinical factors associated with both the presence and persistence of anemia.

## Materials and methods

Study design and setting

This was a retrospective cohort study conducted at the Department of Pediatrics, Armed Forces Hospital - Southern Region (AFHSR), a tertiary referral center serving the southern region of Saudi Arabia.

The study evaluated pediatric patients aged 1-18 years, diagnosed with CD over an eight-year period, from January 2016 to December 2023.

Study population

The study included pediatric patients diagnosed with CD between January 2016 and December 2023. Diagnosis was established in accordance with contemporaneous European Society for Paediatric Gastroenterology, Hepatology and Nutrition (ESPGHAN) guidelines applicable at the time of diagnosis. Accordingly, cases diagnosed prior to 2020 were confirmed by histopathological evidence from small intestinal biopsy, whereas diagnoses made from 2020 onward were based on either histopathology or serology alone in accordance with updated ESPGHAN recommendations [13]. All diagnoses were made by pediatric gastroenterologists, and all eligible patients during the study period were included using a total sampling approach.

Inclusion criteria

Children diagnosed with CD during the study period were eligible for inclusion if the diagnosis was confirmed by intestinal biopsy and/or serologic testing in accordance with ESPGHAN guidelines. Participants were required to have baseline hematologic data available at the time of diagnosis and follow-up hematologic data at both one year and two years following initiation of a GFD.

Exclusion criteria

Patients were excluded if they had alternative causes of anemia, including but not limited to hemoglobinopathies, inflammatory bowel disease, or chronic kidney disease. Additional exclusion criteria included incomplete medical records that precluded accurate assessment of anemia status or follow-up outcomes, and documented non-adherence to a GFD during the follow-up period.

Data collection

Data were extracted retrospectively from electronic medical records using a standardized data collection form. Collected variables included demographic characteristics (age at diagnosis, sex), clinical features at presentation, diagnostic modality (biopsy or serology), syndromic status, hospital admission history, and reported symptoms at diagnosis and follow-up.

Hematologic parameters were reviewed to determine the presence of IDA at diagnosis and during follow-up. Iron therapy data, including type of iron preparation, dosing frequency, and duration of treatment, were recorded when applicable. Follow-up outcomes focused on resolution or persistence of IDA at one year and two years after initiation of a GFD.

Definitions

IDA was defined based on age-specific hemoglobin reference ranges and laboratory findings consistent with iron deficiency, as documented in patient medical records at the time of CD diagnosis. Hemoglobin thresholds were interpreted according to standard pediatric age-defined norms used in routine clinical practice at the study center. Persistence of IDA was defined as continued evidence of anemia on follow-up laboratory evaluation despite adherence to a GFD.

Iron therapy data were extracted from medical records and included documentation of treatment type and duration. All patients who received iron supplementation were treated with oral iron preparations; no patients received intravenous iron therapy during the study period. Iron therapy duration was categorized according to documented treatment length.

Age at diagnosis was categorized into four groups: 0-5 years, 6-10 years, 11-14 years, and ≥15 years. Syndromic status was determined based on clinical documentation of associated genetic or congenital syndromes.

Outcome measures

The primary outcome was the prevalence of IDA at the time of CD diagnosis. Secondary outcomes included persistence of anemia at one year and two years following initiation of a GFD and identification of demographic and clinical factors associated with the presence and persistence of anemia.

Statistical analysis

Descriptive statistics were used to summarize demographic and clinical characteristics. Categorical variables were presented as frequencies and percentages. Associations between patient characteristics and IDA were assessed using chi-square or Fisher’s exact tests, as appropriate.

Multivariable logistic regression analyses were performed to identify independent predictors of IDA at diagnosis and predictors of persistent anemia at one-year follow-up. Adjusted odds ratios (aORs) with corresponding 95% confidence intervals (CIs) were reported. Statistical significance was defined as a two-sided p-value <0.05. All statistical analyses were conducted using IBM SPSS Statistics for Windows, version 26.0 (IBM Corp., Armonk, NY, USA).

Ethical considerations

The study was approved by the Research Ethics Committee of the Medical Services Department for the Armed Forces. Given the retrospective nature of the study, informed consent was waived. Patient confidentiality was strictly maintained through anonymization of data, and no direct patient contact or intervention occurred.

## Results

Study population and baseline characteristics

A total of 97 children with CD were included in the analysis. Females comprised 59 (60.8%) of the cohort, while males accounted for 38 (39.2%). The most common age at diagnosis was 6-10 years, observed in 44 children (45.4%), followed by 11-14 years in 35 (36.1%). Fewer patients were diagnosed at 0-5 years (n = 13, 13.4%) or at ≥15 years of age (n = 5, 5.2%).

Recurrent hospital admissions were infrequent, with 94 patients (96.9%) reporting no recurrence. Similarly, 91 children (93.8%) had no hospital admissions during the preceding 12 months, and 88 (90.7%) reported no average annual hospital admissions.

Diagnosis was predominantly established according to the ESPGHAN criteria [13]. Histological confirmation by intestinal biopsy was performed in 79 patients (81.4%), while 14 patients (14.4%) were diagnosed based on serological criteria alone. The diagnostic method was not documented in four cases (4.1%). Children with undocumented diagnostic modality were included in overall descriptive analyses but excluded from comparisons by diagnostic method. No significant difference in the prevalence of IDA was observed between biopsy-confirmed and serology-only diagnoses. Most children were non-syndromic (n = 93, 95.9%), with only four patients (4.1%) identified as having an associated syndrome (Table 1).

**Table 1:** Baseline characteristics of the study population (n = 97). ESPGHAN: European Society for Paediatric Gastroenterology, Hepatology and Nutrition.

Variable	Category	Number	Percentage
Gender	Female	59	60.8
	Male	38	39.2
Age at diagnosis of celiac disease	0-5 years	13	13.4
	6-10 years	44	45.4
	11-14 years	35	36.1
	≥15 years	5	5.2
Recurrent admission	No	94	96.9
	Yes	3	3.1
Average admissions per year	None	88	90.7
	1 time	5	5.2
	2-3 times	2	2.1
	≥4 times	2	2.1
Admissions in the last 12 months	None	91	93.8
	1	5	5.2
	2	1	1.0
Biopsy/serology (ESPGHAN)	Biopsy	79	81.4
	Serology only	14	14.4
	None	4	4.1
Syndromic child	No	93	95.9
	Yes	4	4.1

Clinical presentation at diagnosis and follow-up

The clinical presentation of CD at diagnosis was heterogeneous (Table 2). Type 1 diabetes mellitus was the most frequently associated condition, identified in 23 patients (23.7%). Short stature was reported in 14 children (14.4%), while diagnosis through asymptomatic screening occurred in 16 cases (16.5%). Notably, 14 patients (14.4%) had no reported symptoms at the time of diagnosis.

**Table 2:** Symptoms of celiac disease (n = 97). FTT: failure to thrive.

	Symptom category	Number	Percentage
At the time of diagnosis	Abdominal pain	3	3.1
	Constipation	2	2.1
	Diarrhea	9	9.3
	Poor weight gain/FTT	8	8.2
	Type 1 diabetes mellitus	23	23.7
	Hypothyroidism	1	1.0
	Short stature	14	14.4
	Food allergy	3	3.1
	Family history of celiac disease	2	2.1
	Screening (asymptomatic)	16	16.5
	No symptoms	14	14.4
	Other	4	4.1
Current symptoms at follow-up	No symptoms	48	49.5
	Screening (asymptomatic follow-up)	24	24.7
	Abdominal pain	3	3.1
	Diarrhea	4	4.1
	Vomiting	4	4.1
	Constipation	1	1.0
	Mixed GI symptoms	4	4.1
	Short stature	10	10.3
	Poor weight gain/FTT	6	6.2
	Allergy/atopy	2	2.1
	Endocrine/other comorbidities	4	4.1

Classical gastrointestinal manifestations were less common, including diarrhea in nine patients (9.3%), poor weight gain or failure to thrive in eight (8.2%), abdominal pain in three (3.1%), and constipation in two (2.1%). Other presenting features included food allergy in three patients (3.1%), a positive family history of CD in two (2.1%), hypothyroidism in one (1.0%), and miscellaneous symptoms in four (4.1%).

At one-year follow-up, clinical status data were available for the full cohort (n = 97). Nearly half of the patients remained asymptomatic (n = 48, 49.5%), while an additional 24 patients (24.7%) were undergoing routine asymptomatic screening. Persistent clinical features were reported in a minority of patients and included short stature (n = 10, 10.3%), poor weight gain or failure to thrive (n = 6, 6.2%), diarrhea (n = 4, 4.1%), vomiting (n = 4, 4.1%), mixed gastrointestinal symptoms (n = 4, 4.1%), abdominal pain (n = 3, 3.1%), and constipation (n = 1, 1.0%).

At the two-year follow-up, clinical status assessment was available for the same cohort. The overall symptom profile remained similar, with the majority of patients asymptomatic or identified through routine screening, and persistent gastrointestinal or growth-related symptoms continuing to be reported in a small proportion of patients.

Allergic or atopic manifestations were present in two children (2.1%), and other endocrine or medical comorbidities were reported in four (4.1%).

Prevalence of iron deficiency anemia and iron therapy

IDA was identified at the time of CD diagnosis in 25 children (25.8%). Among children with IDA, most were diagnosed between six and 10 years of age (n = 14, 56.0%), followed by those aged 11-14 years (n = 7, 28.0%). Smaller proportions were diagnosed at 1-5 years (n = 3, 12.0%) and at ≥15 years of age (n = 1, 4.0%).

Iron supplementation was not administered to the majority of children with CD. Among those treated for IDA, ferric salts were prescribed in seven children (7.2%), ferrous salts in two (2.1%), and ferrous sulfate combined with folic acid in two (2.1%). Iron therapy was administered orally using standard pediatric weight-based dosing, most commonly approximately 3 mg/kg/day of elemental iron.

Among the 11 children who received iron therapy, once-daily dosing was the most common regimen (n = 10, 90.9%), while only one patient (9.1%) received twice-daily dosing. The duration of iron therapy was predominantly three months (n = 22, 88.0%), with fewer patients treated for six months (n = 2, 8.0%) or nine months (n = 1, 4.0%).

Among the 25 children with IDA at diagnosis, follow-up hematologic data were available for all patients at both one-year and two-year time points, with no loss to follow-up. At one year following initiation of a GFD, IDA had resolved in 17 children (68.0%), while eight patients (32.0%) continued to exhibit persistent anemia. By two years post-diet initiation, resolution increased to 22 patients (88.0%), with persistent IDA observed in only three children (12.0%) (Table 3).

**Table 3:** Iron deficiency anemia (IDA) and iron therapy characteristics.

	IDA status	Number	Percentage
Prevalence of iron deficiency anemia (IDA) at diagnosis	No IDA	72	74.2
	Yes (IDA present)	25	25.8
Age at diagnosis among children with IDA (n = 25)	1-5 years	3	12.0
	6-10 years	14	56.0
	11-14 years	7	28.0
	≥15 years	1	4.0
Iron therapy characteristics (n = 25)	No treatment	86	88.7
	Ferric salts	7	7.2
	Ferrous salts	2	2.1
	Ferrous sulfate + folic acid	2	2.1
Frequency of iron therapy (n = 11)	Once daily	10	90.9
	Twice daily	1	9.1
Duration of iron therapy (n = 25)	3 months	22	88.0
	6 months	2	8.0
	9 months	1	4.0
Persistence at 1 year (n = 25)	No (resolved)	17	68.0
	Yes (persistent IDA)	8	32.0
Persistence at 2 years (n = 25)	No (resolved)	22	88.0
	Yes (persistent IDA)	3	12.0

Factors associated with iron deficiency anemia

No statistically significant association was observed between age at diagnosis and the presence of IDA. The highest proportion of IDA was observed among children diagnosed at 6-10 years of age (14/44, 31.8%), followed by those aged 11-14 years (8/35, 22.9%), while lower proportions were noted in children aged 0-5 years (2/13, 15.4%) and ≥15 years (1/5, 20.0%) (p = 0.611).

Gender was not significantly associated with IDA status, with comparable prevalence among females (16/59, 27.1%) and males (9/38, 23.7%) (p = 0.706).

Growth-related parameters also showed no significant associations with IDA. Anemia was present in 22 of 81 children without short stature (27.2%) compared with three of 16 children with short stature (18.8%) (p = 0.482). Similarly, IDA was observed in 24 of 93 children without failure to thrive, defined based on weight-for-age or body mass index below the 5th percentile for age and sex, compared with one of four children meeting criteria for failure to thrive (25.0%) (p = 0.971).

Although a higher proportion of syndromic children had IDA (2/4, 50.0%) compared with non-syndromic children (23/93, 24.7%), this difference did not reach statistical significance (p = 0.258) (Table 4).

**Table 4:** Association between patient characteristics and presence of iron deficiency anemia (IDA) in children with celiac disease. FTT: failure to thrive. Values are presented as n (%). * P-values represent comparisons between groups.

Variable	Category	No IDA, n (%)	IDA, n (%)	Total	p-value*
Age at diagnosis	0-5 years	11 (84.6%)	2 (15.4%)	13	0.611
	6-10 years	30 (68.2%)	14 (31.8%)	44	
	11-14 years	27 (77.1%)	8 (22.9%)	35	
	≥15 years	4 (80.0%)	1 (20.0%)	5	
Gender	Female	43 (72.9%)	16 (27.1%)	59	0.706
	Male	29 (76.3%)	9 (23.7%)	38	
Short stature	No	59 (72.8%)	22 (27.2%)	81	0.482
	Yes	13 (81.3%)	3 (18.8%)	16	
Poor weight gain/FTT	No	69 (74.2%)	24 (25.8%)	93	0.971
	Yes	3 (75.0%)	1 (25.0%)	4	
Syndromic child	No	70 (75.3%)	23 (24.7%)	93	0.258
	Yes	2 (50.0%)	2 (50.0%)	4	

Multivariable analysis of predictors of iron deficiency anemia

In multivariable logistic regression analysis, none of the examined variables were independently associated with the presence of IDA at the time of CD diagnosis. Compared with children diagnosed at 6-10 years of age, those diagnosed at 0-5 years (aOR = 0.48, 95% CI: 0.12-1.92, p = 0.311), 11-14 years (aOR = 0.65, 95% CI: 0.27-1.53, p = 0.329), or ≥15 years (aOR = 0.72, 95% CI: 0.07-6.64, p = 0.789) showed no statistically significant differences in risk.

Gender was not associated with IDA (aOR = 0.84, 95% CI: 0.34-1.97, p = 0.681). Growth-related indicators, including short stature (aOR = 0.60, p = 0.422) and failure to thrive (aOR = 1.04, p = 0.978), were also not significantly associated with anemia. Although syndromic status showed higher point estimates, this association was not statistically significant (aOR = 3.21, 95% CI: 0.42-24.6, p = 0.241) (Table 5).

**Table 5:** Multivariable logistic regression analysis of predictors of iron deficiency anemia (IDA) in children with celiac disease. FTT: failure to thrive. Hyphens (-) indicate reference categories or model parameters for which estimates are not applicable or not calculated.

Predictor	Category	Adjusted OR	95% CI	p-value
Age at diagnosis	0-5 years	0.48	0.12-1.92	0.311
	11-14 years	0.65	0.27-1.53	0.329
	≥15 years	0.72	0.07-6.64	0.789
	(Ref.: 6-10 years)	-	-	-
Gender	Female (Ref.)	0.84	0.34-1.97	0.681
Short stature	Yes vs. No	0.60	0.16-2.11	0.422
Poor weight gain/FTT	Yes vs. No	1.04	0.11-9.09	0.978
Syndromic child	Yes vs. No	3.21	0.42-24.6	0.241
Constant	-	0.29	-	0.127

Given the modest sample size and number of outcome events, this multivariable analysis was exploratory in nature and should be interpreted cautiously, as the study was not powered to detect small-to-moderate associations.

Predictors of persistent iron deficiency anemia at one-year follow-up

In exploratory multivariable logistic regression analysis, no demographic, clinical, or treatment-related variables were independently associated with persistent IDA at one-year follow-up among children with CD. Age at diagnosis, gender, growth indicators (including short stature and failure to thrive), iron formulation, and duration of iron therapy showed no statistically significant associations with anemia persistence (Table 6).

**Table 6:** Multivariable logistic regression analysis of predictors of persistent IDA at one-year follow-up (n = 25). OR: odds ratio; CI: confidence interval; IDA: iron deficiency anemia; FTT: failure to thrive. Hyphens (-) indicate reference categories or model parameters for which estimates are not applicable or not calculated.

Predictor	Category (Reference)	Adjusted OR	95% CI	p-value
Age at diagnosis of IDA	Ref. = 6-10 years	-	-	-
1-5 years	-	0.62	0.05-6.77	0.703
11-14 years	-	1.28	0.19-7.84	0.791
≥15 years	-	2.10	0.09-48.2	0.615
Gender	Female (Ref.)	1.34	0.22-7.91	0.755
Short stature	Yes vs. No	1.88	0.28-12.6	0.528
Poor weight gain/FTT	Yes vs. No	1.41	0.15-13.3	0.760
Treatment type	Ref. (Combined ferrous + folic acid)	-	-	-
Ferric salts	-	1.92	0.17-21.1	0.591
Ferrous salts	-	0.88	0.05-16.2	0.947
Duration of therapy	Continuous (months)	1.12	0.69-1.82	0.633
Constant	-	0.14	-	0.186

Given the limited number of children with persistent anemia at one year, this analysis was not powered to detect small-to-moderate effects and should be interpreted cautiously.

## Discussion

IDA represents one of the most frequent extraintestinal manifestations of CD in children and may precede or overshadow gastrointestinal symptoms. In this retrospective cohort from a tertiary care center in southern Saudi Arabia, IDA was present in approximately one-quarter of children at the time of CD diagnosis. This finding reinforces the clinical importance of considering CD in the diagnostic evaluation of pediatric anemia, even in the absence of classic gastrointestinal features.

Prevalence of iron deficiency anemia at diagnosis

The observed prevalence of IDA at diagnosis (25.8%) falls within the range reported in pediatric cohorts internationally, although it is lower than figures reported in some earlier studies describing prevalence rates approaching 40-50% [2,5]. This variability likely reflects differences in referral patterns, diagnostic timing, and population-based screening practices. However, this prevalence must be interpreted within the local epidemiological context. Recent data indicate that Saudi Arabia has a high background prevalence of IDA among children and adolescents, estimated at approximately 26-37% in some regional studies [14,15]. Consequently, distinguishing between nutritional anemia caused by dietary insufficiency and that caused by celiac-associated malabsorption is critical in this population.

The relatively lower prevalence in our cohort compared to older international data may also suggest earlier detection of CD in our tertiary setting, potentially capturing patients before severe micronutrient depletion occurs. This aligns with a recent meta-analysis of CD in Saudi Arabia, which suggests a high seroprevalence (approximately 2.7%) and an increasing rate of diagnosis among asymptomatic or non-classical presentations [16]. Notably, IDA in the present cohort was most frequently observed among children diagnosed between six and 10 years of age. While this age group demonstrated the highest proportion of anemia, no statistically significant association was identified between age at diagnosis and anemia status, consistent with prior reports [5,9].

Pathophysiology: beyond malabsorption

The pathophysiology of IDA in CD is multifactorial. While villous atrophy and reduced absorptive surface area in the proximal duodenum are the primary drivers, recent evidence highlights the role of systemic inflammation. Active CD triggers an upregulation of hepatic hepcidin, a peptide that inhibits iron transport by blocking ferroportin [17]. This mechanism mimics the "anemia of chronic disease," effectively trapping iron within macrophages and enterocytes and preventing its utilization for erythropoiesis, even when oral iron is administered [18].

This inflammatory blockade explains why some patients present with "refractory" IDA that fails to respond to standard oral iron therapy. Indeed, clinical guidelines now emphasize that unexplained or refractory iron deficiency is a strong indication for CD screening [13]. Understanding this dual mechanism, i.e., malabsorption plus inflammatory blockade, is essential for managing expectations regarding the speed of hematologic recovery.

Clinical characteristics and lack of predictive factors

No significant associations were identified between IDA and gender, growth impairment, or syndromic status in the present study. Although syndromic children demonstrated a numerically higher proportion of anemia, this difference did not reach statistical significance, likely reflecting the small number of syndromic patients in the cohort. Similar findings have been reported in other pediatric studies, where clinical features alone were insufficient to reliably predict anemia risk at diagnosis [6,10].

Importantly, classic gastrointestinal symptoms were relatively uncommon at presentation, while a substantial proportion of children were diagnosed through screening or presented with non-specific or extraintestinal features. This aligns with the evolving clinical phenotype of pediatric CD, in which silent or atypical presentations predominate [4,11]. These findings further emphasize that IDA may occur independently of gastrointestinal symptom burden and should prompt consideration of CD irrespective of symptom profile.

Response of iron deficiency anemia to gluten-free diet

The majority of children with IDA demonstrated hematologic recovery following the initiation of a GFD. Resolution occurred in over two-thirds of affected patients at one year and increased to nearly 90% by two years. These findings are consistent with longitudinal studies showing progressive improvement in anemia following dietary treatment, reflecting mucosal healing and restoration of intestinal absorptive capacity [9,10].

However, three children (12.0%) continued to exhibit persistent anemia at two years despite adherence to a GFD. This persistence mirrors findings from a large Italian cohort study, which reported that while children generally recover faster than adults, a small percentage of pediatric patients remain anemic years after diagnosis due to slow histological recovery or inadvertent gluten exposure [10]. It is also possible that micronutrient co-deficiencies, such as vitamin B12 or folate deficiency, contribute to persistent hematologic abnormalities in these "non-responders," necessitating a broader nutritional assessment during follow-up [19].

Role of iron supplementation

Most children in this cohort did not receive iron supplementation, and among those treated, oral iron therapy, predominantly once-daily dosing for three months, was the most common approach. The absence of a significant association between iron therapy type or duration and anemia persistence supports existing evidence that dietary treatment alone is sufficient for hematologic recovery in many children with CD [9]. However, iron supplementation remains clinically relevant in selected patients, particularly those with symptomatic or severe anemia.

Clinical implications

These findings have several important clinical implications. First, routine screening for CD should be strongly considered in children presenting with IDA, even in the absence of gastrointestinal symptoms. This aligns with the 2020 ESPGHAN guidelines, which list chronic IDA as a key extraintestinal indication for serologic testing [13]. Second, while most children experience gradual resolution of anemia following a GFD, continued hematologic monitoring is warranted, particularly during the first two years of treatment. Finally, for the minority of patients with persistent anemia, clinicians should evaluate for dietary adherence, co-existing micronutrient deficiencies, or potential refractory iron malabsorption that may require alternative iron formulations.

In conclusion, IDA affected approximately one-quarter of children at the time of CD diagnosis in this cohort. Most cases resolved following adherence to a GFD, with continued improvement over time. These findings underscore the importance of systematic anemia screening at diagnosis and structured follow-up in children with CD.

Strengths and limitations

The strengths of this study include its well-defined pediatric cohort, longitudinal follow-up, and comprehensive assessment of anemia outcomes within a tertiary care setting. However, several limitations should be acknowledged. First, the relatively small sample size may have limited the statistical power to detect modest associations, particularly in multivariable analyses. Second, the retrospective study design may introduce information and documentation bias.

Third, the diagnosis of CD was established using a combination of serological criteria and histopathological confirmation, reflecting changes in diagnostic guidelines over time; this heterogeneity may have influenced subgroup analyses. Fourth, although follow-up data were available for most patients, not all children had complete hematologic assessments at both one-year and two-year follow-up intervals, which may have affected estimates of anemia persistence and resolution.

Finally, certain clinical variables, such as slow weight gain and failure to thrive, were based on documentation in the medical record and may not have been uniformly defined or consistently quantified across all patients. Additionally, histological severity and iron metabolism biomarkers were not uniformly available, precluding more granular analysis of mucosal recovery and iron regulation. These limitations should be considered when interpreting the findings.

## Conclusions

IDA is a common extraintestinal manifestation of CD in children, affecting approximately one quarter of patients at the time of diagnosis in this cohort. Most affected children demonstrated gradual hematologic recovery following initiation of a GFD, with continued improvement over time; however, a subset showed delayed resolution, underscoring the need for structured and ongoing follow-up.

No demographic, clinical, or treatment-related factors were independently associated with either the presence or persistence of anemia, reflecting the heterogeneous nature of IDA in pediatric CD. These findings highlight the importance of maintaining a high index of suspicion for CD in children presenting with IDA, regardless of gastrointestinal symptoms, and support early diagnosis, adherence to a GFD, and individualized hematologic monitoring to optimize long-term outcomes.
